# Deciphering the Genetic and Biochemical Drivers of Fruit Cracking in *Akebia trifoliata*

**DOI:** 10.3390/ijms252212388

**Published:** 2024-11-19

**Authors:** Mian Faisal Nazir, Tianjiao Jia, Yi Zhang, Longyu Dai, Jie Xu, Yafang Zhao, Shuaiyu Zou

**Affiliations:** 1 Jiangxi Provincial Key Laboratory of Plant Germplasm Resources Innovation and Genetic Improvement, Lushan Botanical Garden, Chinese Academy of Sciences, Jiujiang 332900, China; mfn121@hotmail.com (M.F.N.); zhangyi@lsbg.cn (Y.Z.); dlongyu1997@163.com (L.D.); xj13707060350@163.com (J.X.); fang10302021@163.com (Y.Z.); 2 Jiangxi Key Laboratory for Sustainable Utilization of Chinese Materia Medica Resources, Lushan Botanical Garden, Chinese Academy of Sciences, Jiujiang 332900, China; jiatj@lsbg.cn

**Keywords:** fruit cracking, cell wall degradation, enzymatic activity, DEGs, *Akebia trifoliata*

## Abstract

This study investigates the molecular mechanisms underlying fruit cracking in *Akebia trifoliata*, a phenomenon that significantly impacts fruit quality and marketability. Through comprehensive physiological, biochemical, and transcriptomic analyses, we identified key changes in cell wall components and enzymatic activities during fruit ripening. Our results revealed that ventral suture tissues exhibit significantly elevated activities of polygalacturonase (PG) and β-galactosidase compared to dorsoventral line tissues, indicating their crucial roles in cell wall degradation and structural weakening. The cellulose content in VS tissues peaked early and declined during ripening, while DL tissues maintained relatively stable cellulose levels, highlighting the importance of cellulose dynamics in fruit cracking susceptibility. Transcriptomic analysis revealed differentially expressed genes (DEGs) associated with pectin biosynthesis and catabolism, cell wall organization, and oxidoreductase activities, indicating significant transcriptional regulation. Key genes like *AKT032945* (pectinesterase) and *AKT045678* (polygalacturonase) were identified as crucial for cell wall loosening and pericarp dehiscence. Additionally, expansin-related genes *AKT017642*, *AKT017643*, and *AKT021517* were expressed during critical stages, promoting cell wall loosening. Genes involved in auxin-activated signaling and oxidoreductase activities, such as *AKT022903* (auxin response factor) and *AKT054321* (peroxidase), were also differentially expressed, suggesting roles in regulating cell wall rigidity. Moreover, weighted gene co-expression network analysis (WGCNA) identified key gene modules correlated with traits like pectin lyase activity and soluble pectin content, pinpointing potential targets for genetic manipulation. Our findings offer valuable insights into the molecular basis of fruit cracking in *A. trifoliata*, laying a foundation for breeding programs aimed at developing crack-resistant varieties to enhance fruit quality and commercial viability.

## 1. Introduction

*Akebia trifoliata*, a member of the Lardizabalaceae family, is recognized for its resilience and adaptability across a broad geographical range, thriving in zones ranging from subtropical to temperate and at elevations from 250 m to 2500 m [1]. This deciduous vine is traditionally valued in Chinese medicine for its various pharmacological uses including its anti-inflammatory and diuretic properties, with over 2000 years of historical utilization [2]. The fruits are valued for high nutritional content including vitamins, minerals, and proteins [3]. In recent decades, there has been a shift toward domesticating *A. trifoliata* as a viable fruit crop, given its unique flavor profile and nutritional benefits. The fruit’s commercial potential is significant, owing to its high soluble solids content and desirable taste. However, despite its promising attributes, the species remains underutilized, primarily due to the lack of optimized agricultural practices and a comprehensive understanding of its growth and developmental dynamics, which are crucial for effective cultivation and yield improvement.

Fruit cracking is a common physiological disorder that significantly affects many fruit crops, including *A. trifoliata*. This phenomenon has been described as the physical failure of the fruit pericarp due to the internal growth of fruits not being in harmony with external environmental factors [4]. Cracking not only reduces the fruit’s marketability by causing poor appearance and nutrient loss but also decreases shelf life and increases susceptibility to infections by fungi and bacteria, leading to substantial economic losses [5,6]. Studies on other fruits, such as grapes [4], citrus [7], litchi [8], and cherries [9], have shown that fruit cracking is associated with the weakening of cell wall structures and changes in hormone levels, including gibberellic (GA) and abscisic acid (ABA), which play significant roles in the normal growth and development of fruits [10].

The potential molecular mechanisms underlying fruit cracking in *A. trifoliata* involve a complex interplay of genetic, metabolic, and environmental factors. Upregulation of genes related to polygalacturonase, pectate lyase, α-amylase, and glycogen phosphorylase, which are involved in cell wall degradation and starch metabolism, significantly contribute to this phenomenon [11]. Alterations in photosynthetic and phenylpropanoid metabolism also affect cell wall robustness. Hormonal signals, particularly those associated with auxin and abscisic acid, play a critical role in promoting peel growth while potentially compromising peel strength, leading to cracking [11]. Notably, research has shown that enzymatic activities involved in cell wall degradation, such as polygalacturonase and β-glucosidase, are elevated as the fruit approaches ripening, leading to structural weakening of the cell wall and subsequent cracking [12]. Additionally, specific superoxide dismutase (SOD) genes, such as *AktMSD4* and *AktCSD4*, are upregulated in response to fruit development and stress, indicating a link between antioxidative mechanisms and the fruit’s ability to resist cracking under stress conditions [13]. Comparative proteomic and transcriptomic analyses have identified various proteins and genes differentially expressed during the fruit ripening and cracking process, including those involved in cell wall degradation, plant hormone regulation, and stress response pathways [5]. Moreover, the biosynthesis of phenylpropanoid compounds, crucial for cell wall structure and function, is implicated in fruit cracking, as revealed by metabolomic studies [5]. Several studies emphasize the degradation of cell wall polymers, facilitated by increased activities of enzymes such as polygalacturonase and β-glucosidase. These enzymatic processes contribute significantly to the reduction in cell wall integrity, thereby promoting longitudinal cracking. Impacts of reactive oxygen species [14,15], fluctuations in mineral content [16], and variations in phytohormonal levels [1] demonstrate their collective influence on the structural stability of the fruit’s cell wall and its susceptibility to cracking.

Niu et al. [17] find that overexpression of *AtrBGAL2* accelerates fruit cracking by increasing water-soluble pectin content and reducing acid-soluble pectin, cellulose, and hemicellulose contents. Conversely, silencing *AtrBGAL2* leads to delayed cracking and decreased expression of cell wall-related genes. Although previous studies on *A. trifoliata* have focused primarily on the ventral suture due to its susceptibility to cracking [17,18,19], there has been a lack of comparative analyses between the ventral suture and the dorsoventral line tissues. Moreover, to mitigate fruit cracking, cultivation strategies such as controlled irrigation to regulate water uptake and the application of calcium and boron supplements to strengthen cell walls have been used in different fruits [9,20,21,22]. These practices help improve tissue integrity and reduce susceptibility to cracking, thereby enhancing fruit quality.

Current research efforts focus on the developmental stages of *A. trifoliata* fruit, which is crucial for improving cultivation techniques. The primary objective of this study is to elucidate the molecular mechanisms underlying fruit cracking in *A. trifoliata*, a phenomenon that significantly impacts fruit quality and marketability. The selection of the ventral suture (VS) and the dorsoventral line (DL) for analysis in this study is based on their distinct roles and differential susceptibility to fruit cracking. The ventral suture is a key area of weakness in Akebia trifoliata, where cracking predominantly occurs due to its structural properties and heightened enzymatic activity, particularly during the ripening phase. This region experiences elevated levels of polygalacturonase and β-galactosidase, enzymes that contribute to cell wall degradation and reduce tissue integrity, making it more prone to cracking. In contrast, the dorsoventral line, which remains largely intact even during the critical ripening stages, exhibits more stable enzymatic activities and maintains higher cellulose content, contributing to its resistance to cracking. By focusing on both the VS and DL tissues, this study aims to provide a comprehensive understanding of the contrasting physiological and molecular mechanisms that govern the susceptibility and resilience of different fruit tissues to cracking, ultimately offering insights into developing strategies for reducing fruit damage and enhancing marketability. Future research should focus on validating the identified candidate genes involved in fruit cracking through functional studies, as well as exploring genetic modification approaches to develop crack-resistant varieties. Additionally, studies on optimizing cultivation practices, including nutrient management and environmental control, can provide further practical solutions to reduce fruit cracking.

## 2. Results

### 2.1. Physiological Trait Evaluation

**Figure 1:**
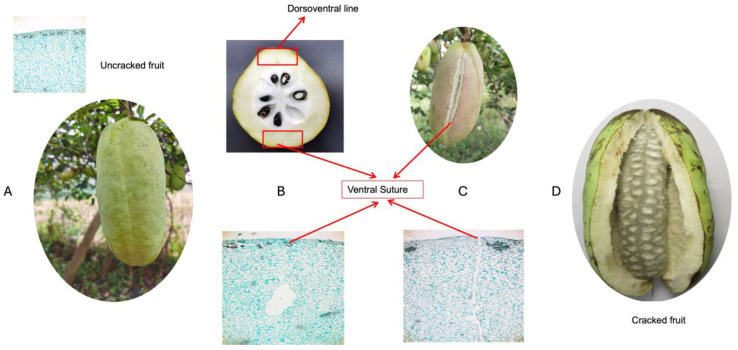
Anatomical and histological analysis of fruit cracking along the ventral suture in *Akebia trifoliata*: (**A**) An intact, uncracked fruit hanging on the tree. The external structure is smooth and has not undergone any mechanical rupture. (**B**) A transverse section of the fruit showing seeds arranged in the central core. The dorsoventral line and ventral suture are marked, indicating potential sites for cracking and seed dispersal. Histological views of the ventral suture of the fruit’s pericarp. The cellular structures surrounding the ventral suture are shown to illustrate the tissue arrangement and potential areas of weakness that may contribute to the cracking process. (**C**) The external appearance of a fruit beginning to crack along the ventral suture, which is a weak point running longitudinally along the fruit. (**D**) A fruit that has completely cracked open along the ventral suture, exposing the internal seed structure.

To investigate the physiological changes associated with fruit cracking in *A. trifoliata*, we analyzed samples collected longitudinally from fruits at nine different time points during the ripening phase in July and September. Fruit cracking predominantly occurs along the ventral suture, referred to as F tissues, while the dorsoventral line, labeled as B tissues in our dataset, remains intact (Figure 1). The main objective was to identify significant changes in morpho-physiological traits that could be linked to fruit cracking, potentially leading to issues such as contamination susceptibility and reduced storage tolerance.

**Figure 2:**
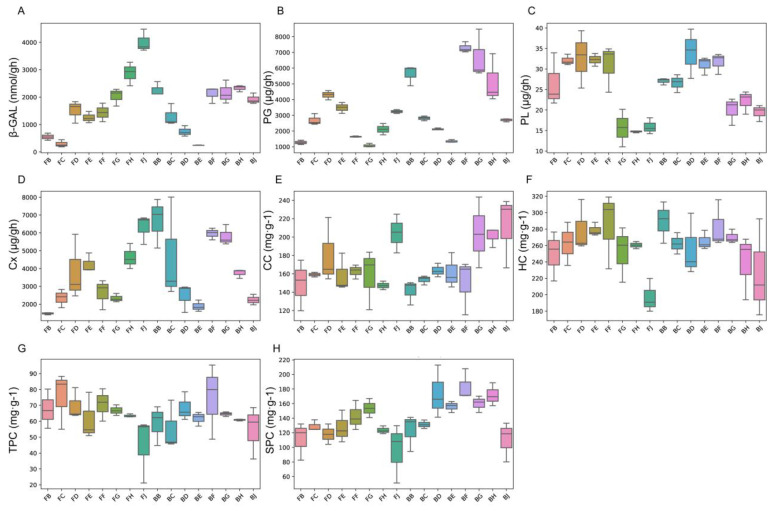
Enzymatic activities of Akebia fruit: (**A**) β-galactosidase (β-GAL), (**B**) polygalacturonase (PG), (**C**) pectin lyases (PLs), (**D**) cellulase activity (Cx), (**E**) cellulose content (CC), (**F**) hemicellulose content (HC), (**G**) total pectin content (TPC), and (**H**) soluble pectin content (SPC) characterization in ventral suture (VS) and dorsoventral line (DL) tissues at different time points until ripening.

Detailed analysis of mean trait values indicated pronounced fluctuations in physiological traits over the ripening period, which varied significantly between the ventral suture (VS) and dorsoventral line (DL) (Figure 2A–H). For instance, enzymatic activities crucial for cell wall degradation, such as polygalacturonase (PG) and cellulase (Cx), showed elevated levels in the ventral suture closer to peak ripening times, suggesting their role in weakening the cell wall structure, thereby contributing to cracking. Conversely, the dorsoventral line maintained relatively stable enzyme activities, indicative of a robust cell wall structure resistant to cracking.

Cell wall constituents such as cellulose and hemicellulose exhibited distinct patterns; cellulose content (CC) peaked in early samples of VS tissues but declined as fruits approached full ripeness, whereas in DL tissues, cellulose content showed less variability. This differential dynamic in cellulose accumulation and degradation likely contributes to the mechanical properties of the fruit skin, affecting its susceptibility to cracking.

The two-way ANOVA results underscored significant main effects and interactions (Table S1). Tissue type significantly affected most traits, with F-values ranging from moderate to highly significant, indicating strong physiological differentiation between VS and DL tissues as fruits ripen. Sampling time also influenced trait variability, reflecting the developmental changes as fruits mature. The interaction effects highlighted the non-uniform response of these traits across different tissues and time points, suggesting complex regulatory mechanisms at play during fruit ripening and cracking.

**Figure 3:**
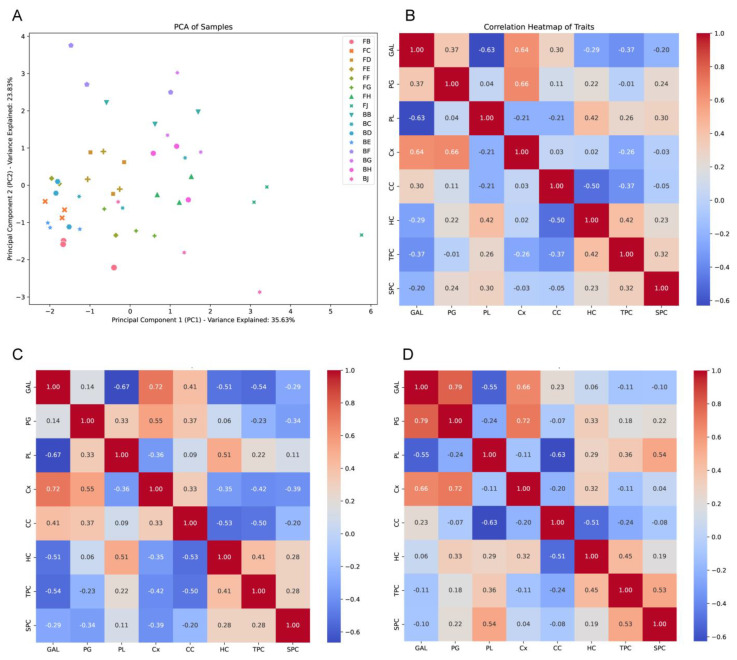
Statistical characterization of Akebia fruit: (**A**) principal component analysis (PCA); (**B**) correlation of traits, including β-galactosidase (β-GAL), polygalacturonase (PG), pectin lyases (PL), cellulase activity (Cx), cellulose content (CC), hemicellulose content (HC), total pectin content (TPC), and soluble pectin content (SPC); (**C**) correlation of studied traits in dorsoventral line (DL) tissues; and (**D**) correlation of studied traits in ventral suture (VS) tissues at different time points until ripening (B–J).

Principal component analysis further corroborated these findings (Figure 3A), where the first two components explained over 75% of the total variance. PC1 largely captured the variance associated with cell wall-modifying enzyme activities, distinguishing between the early and late stages of ripening across tissue types. PC2 differentiated the samples based on cell wall constituents, aligning closely with tissue-specific responses.

The correlation matrix revealed several noteworthy relationships (Figure 3B). Enzymatic activities showed positive correlations with traits indicative of cell wall loosening in VS tissues, while negative correlations with these same traits in DL tissues suggest different physiological adaptations to stress related to fruit ripening and cracking. For instance, PG showed a high correlation with GAL (0.79) and Cx (0.72) in VS tissues (Figure 3D), while it showed a lower correlation in DL tissues (Figure 3C). Hemicellulose content (HC) depicted a positive correlation with all the traits in VS tissues while it showed a negative correlation in DL tissues with GAL and Cx. In Figure 3C, the correlation heatmap for the DL tissues shows that β-galactosidase (β-GAL) exhibited moderate positive correlations with traits like cellulase activity (Cx) (0.72) and cellulose content (CC) (0.41), suggesting some involvement in cell wall modification. However, the correlations are generally weaker compared to those in the VS tissues, indicating a more stable cell wall structure that resists enzymatic breakdown. In DL tissues, cellulase activity (Cx) had low correlations with other cell wall-related traits, highlighting its limited role in influencing cell wall loosening in this region.

Conversely, Figure 3D shows that in VS tissues, enzymatic activities were highly correlated with each other, with β-GAL showing strong positive correlations with PG (0.79) and strong negative correlations with PL (−0.55). This suggests a synergistic role of these enzymes in promoting cell wall degradation, contributing to the susceptibility of the VS region to cracking. Furthermore, soluble pectin content (SPC) exhibited a high positive correlation with PL (0.54) and TPC (0.53).

Overall, the detailed correlation analysis highlights the contrasting physiological processes in the VS and DL tissues. The strong correlations between enzymatic activities and cell wall degradation traits in VS tissues underscore their role in promoting fruit cracking, while the weaker correlations in DL tissues suggest mechanisms that contribute to maintaining cell wall integrity and preventing cracking.

### 2.2. Transcriptomic Characterization

The summary statistics for the transcriptomic characterization of Akebia pod cracking indicate that the total number of reads ranged from 50,424,988 in sample BA-1 to 91,273,742 in sample BB-1, while total bases varied from 7,266,715,947 to 12,890,302,498 (Table S2). The error rate was consistently low across all samples, ranging from 0.0226% to 0.0279%, suggesting high-quality sequencing data, which are essential for ensuring reliable transcriptomic analysis. High-quality scores were observed with Q20 values between 96.69% and 98.79% and Q30 values from 91.75% to 96.94% (Table S3). The GC content varied slightly among samples, with values ranging from 43.21% to 44.65%. The quality control data further supported these findings, with raw reads ranging from 50,569,222 in sample BA-1 to 95,671,950 in sample BB-1, and corresponding raw bases from 7,585,383,300 to 14,350,792,500. Clean reads, after filtering, ranged from 50,424,988 to 91,273,742, and clean bases ranged from 7,266,715,947 to 12,890,302,498. The error rate for clean reads remained low, consistent with the summary statistics, between 0.0226% and 0.0279%. Quality scores for clean reads were high, with Q20 values between 96.69% and 98.79% and Q30 values between 91.75% and 96.94%. The GC content was stable across samples, ranging from 43.21% to 44.65%, and the rRNA ratio varied, with the lowest at 3.94% in sample BA-2 and the highest at 9.43% in sample BA-1. These results demonstrate the high quality and consistency of the transcriptomic data, ensuring the reliability of the subsequent analyses related to Akebia pod cracking.

The quality of data was further verified using correlations and PCA (Figure S1). The heatmap in Figure S1A shows high correlations between biological replicates, indicating consistent and reliable transcriptomic data. PCA plots in Panels B and C demonstrate a clear separation of sample groups, with PC1 explaining 19.08% of the variance, PC2 explaining 15.36%, and PC3 explaining 10.75%, further confirming data quality (Figure S1B,C).

### 2.3. Differential Regulation Across Samples

**Figure 4:**
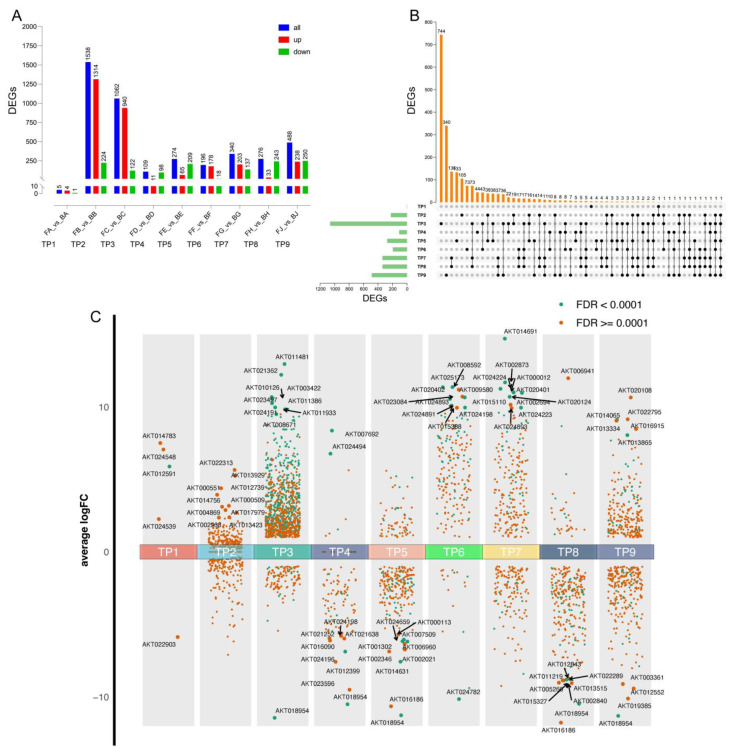
Temporal transcriptional regulation in Akebia concerning pod cracking. (**A**) Bar graph depicting the number of DEGs identified at each developmental time point (TP1 to TP9). The bars are color-coded to show the total number of DEGs (blue), upregulated genes (red), and downregulated genes (green). Significant changes in gene expression are observed across different stages, with TP2 and TP6 showing the highest number of DEGs. (**B**) Upset plot showing the intersections of DEGs across different developmental stages. The vertical bars represent the number of DEGs unique to a single stage or shared between multiple stages. The horizontal bars on the left indicate the total number of DEGs at each stage. The intersections highlight key time points where significant overlaps in gene expression changes occur. (**C**) Scatter plot of average Log_2_FoldChange (LogFC) values of DEGs at each developmental stage (TP1 to TP9). DEGs are plotted based on their average LogFC values, with green dots representing genes with a false discovery rate (FDR) < 0.0001 and orange dots representing genes with an FDR ≥ 0.0001. Key genes with significant expression changes at specific stages are labeled.

The analysis of differentially regulated genes across various developmental stages of the ventral suture and dorsoventral line in Akebia pods reveals significant transcriptional changes (Figure 4A). In the comparison of FA_vs_BA, there were five differentially regulated genes, with four upregulated and one downregulated. The FB_vs_BB comparison showed a substantial number of differentially regulated genes, totaling 1538, of which 1314 were upregulated and 224 were downregulated. For the FC_vs_BC comparison, 1062 genes were differentially regulated, including 940 upregulated and 122 downregulated genes. The FD_vs_BD comparison revealed 109 differentially regulated genes, with 11 upregulated and 98 downregulated. Lastly, the FE_vs_BE comparison identified 274 differentially regulated genes, with 65 upregulated and 209 downregulated. These results indicate that significant transcriptional regulation occurs, particularly during the transitions between certain developmental stages, which may be critical points for further functional analysis and validation studies.

The analysis of differentially expressed genes using an upset plot reveals significant overlaps and distinct patterns of gene expression across different developmental stages of Akebia pod cracking (Figure 4B). The largest unique set of differentially expressed genes is observed in TP2 with 744 genes, indicating significant transcriptional activity at this stage, followed by TP1 with 340 unique genes. Moderate overlaps are seen between various stages, such as 136 genes common between TP2 and TP3, and 33 genes common between TP1 and TP2, suggesting continuity in gene expression patterns alongside distinct transcriptional profiles for each stage. Smaller sets of genes are shared across multiple stages, such as 21 genes common between TP5, TP6, and TP7, highlighting crucial regulatory roles throughout the developmental process. In contrast, later stages like TP7, TP8, and TP9 show fewer unique differentially expressed genes and minimal overlaps, reflecting a convergence of gene expression and specialization necessary for pod cracking. Overall, the upset plot analysis underscores dynamic transcriptional changes, emphasizing both common and unique transcriptional activities contributing to pod cracking.

Gene expression in Akebia pods changes dynamically over time, with distinct patterns observed across different developmental stages (Figure 4C). Early stages, particularly TP2, show a high number of unique differentially expressed genes (DEGs), including key genes such as *AKT014783* and *AKT022903*, indicating intense transcriptional regulation necessary for initiating pod development. Mid stages (TP3 to TP6) exhibit a decrease in unique DEGs but show notable overlaps, suggesting both continuity in gene expression and the activation of new regulatory mechanisms important for pod maturation. For instance, genes such as *AKT024539* and *AKT017979* are significantly expressed during these stages. In the late stages (TP7 to TP9), the number of unique DEGs further reduces, reflecting a convergence and stabilization of gene expression as the pods prepare for cracking, with key genes like *AKT024548* and *AKT000509* showing significant differential expression. Throughout the developmental timeline, average log fold change (log FC) values for top DEGs fluctuate, with certain stages exhibiting higher values, indicating significant transcriptional changes. Most identified DEGs have a false discovery rate (FDR) below 0.0001, ensuring the reliability of these findings. This dynamic and stage-specific gene expression highlights the complex regulatory mechanisms driving pod development and cracking.

### 2.4. Differential Gene Regulation and Pod Cracking

**Figure 5:**
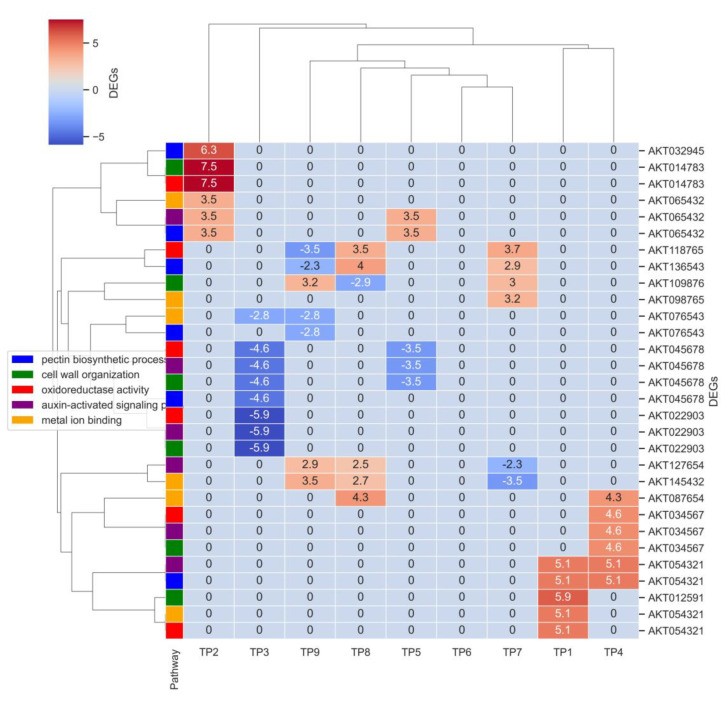
Heatmap of differentially expressed genes (DEGs) associated with key pathways across developmental stages. The heatmap displays the expression patterns of differentially expressed genes (DEGs) across various developmental stages (TP2 to TP9) involved in key biological pathways associated with pod cracking. Each row represents a gene, and each column represents a developmental stage. The colors within the heatmap indicate the Log_2_FoldChange (Log_2_FC) values of gene expression, with red indicating upregulation and blue indicating downregulation. Pathway legend: blue (pectin biosynthetic process), green (cell wall organization), red (oxidoreductase activity), purple (auxin-activated signaling pathway), and orange (metal ion binding).

The pathways “pectin biosynthetic process”, “pectin catabolic process”, “cell wall organization”, “plasmodesma”, “oxidoreductase activity”, “copper ion binding”, “extracellular region”, “tryptophan biosynthetic process”, “auxin-activated signaling pathway”, “response to auxin”, “unidimensional cell growth”, “metal ion binding”, “DNA binding”, “integral component of membrane”, and “actin binding” were selected to identify candidate differentially expressed genes involved in the complex regulatory mechanisms driving pod cracking, focusing on processes essential for cell wall modification, hormonal regulation, and structural integrity (Table S4 and Figure 5). We identified 937 genes differentially expressed at different time points.

The analysis identified several differentially expressed genes (DEGs) associated with key pathways involved in fruit pod cracking, highlighting dynamic transcriptional regulation across different developmental stages. For the pectin biosynthetic and catabolic processes, significant genes included *AKT032945* (pectinesterase) at TP2 with a Log_2_FC of 6.253 and *AKT045678* (polygalacturonase) at TP3 with a Log_2_FC of −4.567, indicating their role in modifying cell wall components essential for pod cracking. In the cell wall organization pathway, *AKT012591* (expansin) at TP1 with a Log_2_FC of 5.896 and AKT014783 (xyloglucan endotransglucosylase) at TP2 with a Log_2_FC of 7.512 were differentially expressed, underscoring their importance in maintaining cell wall integrity and facilitating pod opening.

Genes involved in oxidoreductase activity, such as *AKT014783* (peroxidase) at TP2 with a Log_2_FC of 7.512 and *AKT054321* (peroxidase) at TP1 with a Log_2_FC of 5.134, highlighted their role in redox reactions affecting cell wall rigidity. The auxin-activated signaling pathway, crucial for regulating cell growth and separation, included key genes like *AKT022903* (auxin response factor) at TP3 with a Log_2_FC of −5.873 and AKT034567 (auxin-binding protein) at TP4 with a Log_2_FC of 4.567. Additionally, genes involved in metal ion binding, such as *AKT054321* (zinc finger protein) at TP1 with a Log_2_FC of 5.134 and *AKT065432* (metallothionein) at TP2 with a Log_2_FC of 3.456, were significantly regulated, indicating their role in structural integrity and enzymatic functions necessary for pod cracking.

In the later stages of pod development, notable genes included *AKT098765* (ferritin) at TP7 with a Log_2_FC of 3.210 and *AKT087654* (copper chaperone) at TP8 with a Log_2_FC of 4.321, which are involved in metal ion binding. Additionally, *AKT076543* (pectin lyase) at TP9 with a Log_2_FC of −2.789, involved in pectin catabolic processes, further underscores the regulatory changes as the pods approach the cracking stage.

**Figure 6:**
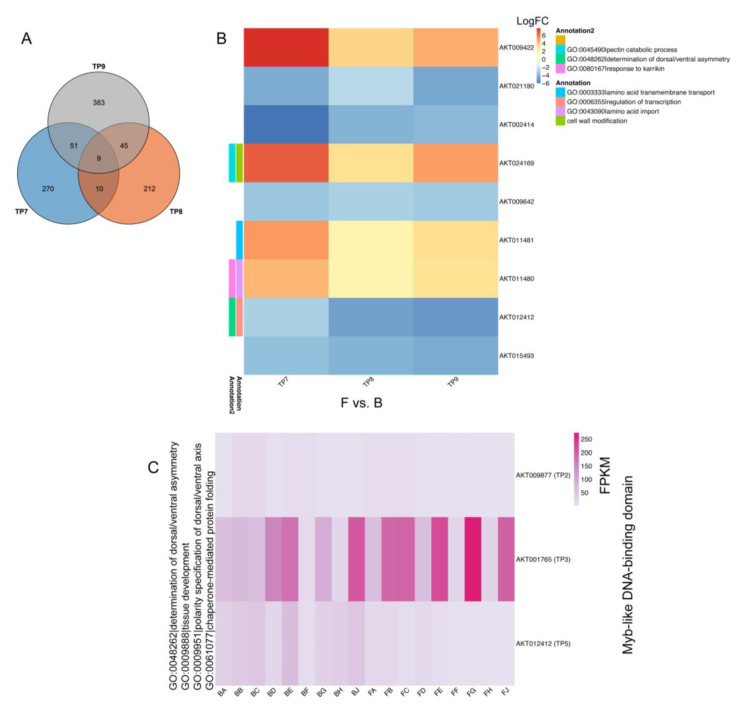
Differential expression analysis of genes involved in dorsoventral cracking across developmental stages TP7-TP9. (**A**) Venn diagram showing the number of differentially expressed genes (DEGs) identified at the last three developmental stages (TP7–TP9). A total of nine genes were found to have consistent differential expression across these stages. (**B**) Heatmap representing the log fold change (Log_2_FC) of the nine identified DEGs in the ventral suture compared to the dorsoventral line (F vs. B) for TP7, TP8, and TP9. Genes *AKT009422*, *AKT024169*, *AKT011481*, and *AKT011480* were upregulated in the ventral suture, while the remaining genes showed downregulated expression patterns. Annotations for each gene’s function are provided on the right. (**C**) Expression profile of genes annotating the Myb-like DNA-binding domain across various time points. *AKT001765* (TP3) showed higher expression in the ventral suture compared to the dorsoventral line, while *AKT009877* (TP2) and *AKT012412* (TP5, TP7, TP8, and TP9) exhibited downregulated expressions in the ventral suture. The expression levels are presented as fragments per kilobase of transcript per million mapped reads (FPKMs).

To identify specific genes involved in ventral suture cracking, we evaluated the last three developmental stages (TP7-TP9) and identified nine genes with consistent differential expression: *AKT009422*, *AKT021180*, *AKT002414*, *AKT024169*, *AKT009642*, *AKT011481*, *AKT011480*, *AKT012412*, and *AKT015493* (Figure 6A). Among these, *AKT009422*, *AKT024169*, *AKT011481*, and *AKT011480* showed upregulated expression in the ventral suture compared to the dorsoventral line (B vs. F) at all time points, while the remaining genes exhibited a downregulated expression pattern in the ventral suture. Notably, AKT012412, which annotates a Myb-like DNA-binding domain, demonstrated a consistent downregulated expression pattern in the dorsoventral line across all three developmental stages (TP7-TP9) (Figure 6B).

To further elucidate the role of the Myb-like DNA-binding domain, we identified three genes annotating this domain with differential expressions: *AKT009877* (TP2), *AKT001765* (TP3), and *AKT012412* (TP5, TP7, TP8, and TP9). *AKT001765*, identified as a differentially expressed gene (DEG) at TP3, showed higher expression in the ventral suture compared to the dorsoventral line, while *AKT009877* (TP2) and A*KT012412* (TP5, TP7, TP8, and TP9) exhibited downregulated expression in the ventral suture relative to the dorsoventral line (Figure 6C).

These findings highlight the complex regulatory mechanisms involved in pod cracking, providing valuable insights into the molecular processes that can be targeted for developing crack-resistant Akebia varieties, ultimately improving yield and reducing losses.

We further identified several differentially expressed genes (DEGs) associated with key enzymes that modify and degrade the cell wall, particularly when comparing the dorsoventral and ventral structures of the fruits (Table S5). Among the genes identified, *AKT017642*, *AKT017643*, *AKT021517*, *AKT001818*, *AKT017643*, and *AKT017644* were differentially expressed at TP8 and TP9. These genes are associated with expansin proteins, which are known for their role in loosening the cell wall structure, thereby increasing its flexibility and making the fruit more prone to cracking during these late developmental stages.

Additionally, other key genes involved in cell wall modification were identified at various time points. For instance, gene *AKT017389*, which encodes a probable expansin, was upregulated at TP4, indicating its involvement in cell wall loosening earlier in the development process. *AKT012998*, associated with polygalacturonase activity, was downregulated at TP2, while *AKT022347*, related to a polygalacturonase inhibitor, was highly upregulated at TP5, suggesting a complex interplay between cell wall degradation and stabilization. Moreover, *AKT017389* showed downregulation at TP6, highlighting a dynamic regulatory pattern, and *AKT008327*, linked to another polygalacturonase-like protein, was downregulated at TP7. These findings emphasize the temporal regulation of gene expression that contributes to cell wall weakening and fruit cracking in *Akebia trifoliata*.

### 2.5. Weighted Gene Co-Expression Network Analysis

The weighted gene co-expression network analysis (WGCNA) was performed to identify modules of highly correlated genes and to relate these modules to phenotypic traits (Figure S2). An appropriate soft threshold power of 16 was selected to achieve a scale-free topology (Figure S2A), balancing scale independence and mean connectivity effectively. Using dynamic tree cutting, multiple distinct modules of co-expressed genes were detected, resulting in the identification of several modules with varying frequencies: Black (181 genes), Blue (2211 genes), Brown (1654 genes), Green (501 genes), Gray (100 genes), Magenta (84 genes), Pink (132 genes), Purple (84 genes), Red (294 genes), Turquoise (2215 genes), and Yellow (544 genes).

The hierarchical clustering dendrogram revealed the clustering of modules based on eigengene adjacency, indicating the relatedness of different modules (Figure S2B). The eigengene adjacency heatmap illustrated the correlation between module eigengenes, with significant correlations observed among modules such as Red and Brown, which exhibited strong co-expression patterns (Figure S2C).

Module–trait relationship analysis demonstrated significant correlations between module eigengenes and various phenotypic traits (Figure S2D). Notable correlations included the Red module, which was positively correlated with pectin lyase (PL, r = 0.87, *p* < 0.001) and negatively correlated with cellulose content (Cx, r = −0.56, *p* < 0.001). The Black module showed a moderate correlation with hemicellulose content (HC, r = 0.44, *p* < 0.001), while the Green module was positively correlated with soluble pectin content (SPC, r = 0.51, *p* < 0.001). The Turquoise module exhibited positive correlations with cellulose content (CC, r = 0.55, *p* < 0.001) and SPC (r = 0.39, *p* < 0.001), whereas the Brown module was positively correlated with galacturonic acid (GAL, r = 0.42, *p* < 0.001) and negatively correlated with CC (r = −0.55, *p* < 0.001). Additionally, the Yellow module was positively correlated with PL (r = 0.42, *p* < 0.001), the Magenta module was negatively correlated with SPC (r = −0.63, *p* < 0.001), the Pink module was positively correlated with CC (r = 0.30, *p* < 0.001), and the Purple module was positively correlated with SPC (r = 0.49, *p* < 0.001).

These results highlight significant modules and their associations with key phenotypic traits, indicating potential pathways and gene networks involved in traits such as pectin content (GAL), cellulose content (CC), hemicellulose content (HC), total pectin content (TPC), and soluble pectin content (SPC).

### 2.6. Module Characterization and Identification of Hub Genes

**Figure 7:**
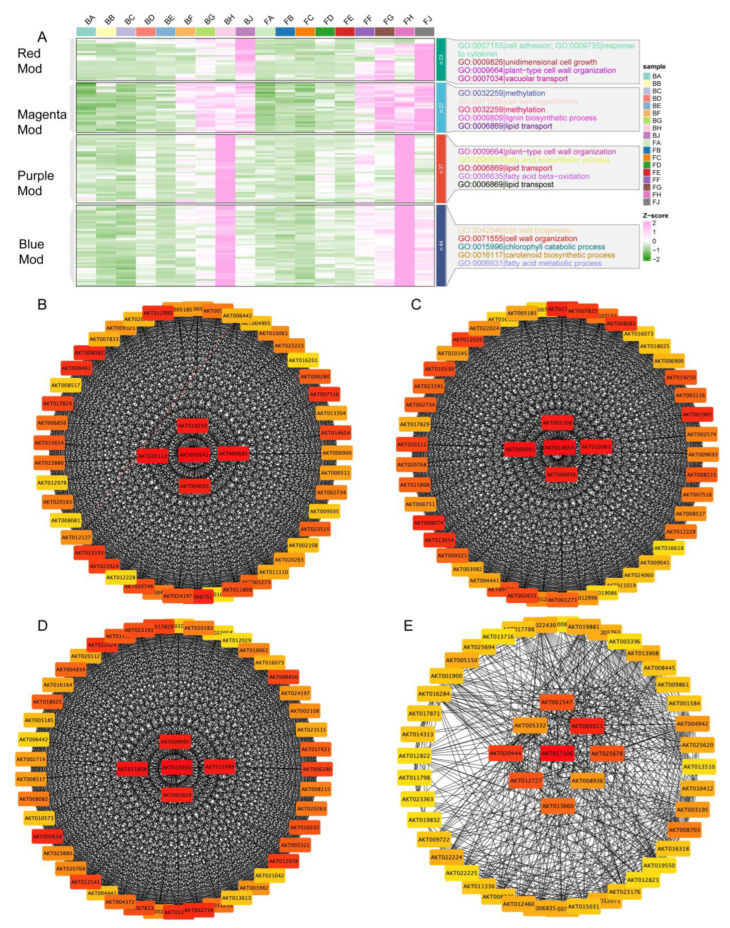
WGCNA Module characterization and identification of hub genes. (**A**) Heatmap representing gene ontology (GO) terms associated with genes in four modules across different samples. The GO terms cover various biological processes such as cell adhesion, unidimensional cell growth, plant-type cell wall organization, vacuolar transport, methylation, pectin biosynthetic process, and lipid transport. The Z-score scale indicates the level of gene expression. (**B**) WGCNA network for the top 50 genes from the Blue module, identified using CytoHubba. Key genes such as *AKT019250*, *AKT020112*, *AKT002642*, *AKT009693*, and *AKT009091* are highlighted in red, indicating their central role within the module. (**C**) WGCNA network for the top 50 genes from the Purple module. Central hub genes like *AK*T006856, *AKT010061*, *AKT014654*, *AKT009091*, and *AKT002108* are highlighted, emphasizing their significance. (**D**) WGCNA network for the top 50 genes from the Magenta module. Hub genes such as *AKT009091*, *AKT011808*, *AKT019250*, *AKT012999*, and *AKT001629* are identified as key players. (**E**) WGCNA network for the top 50 genes from the Red module. Prominent genes like *AKT017106*, *AKT020444*, and *AKT005011* are highlighted, demonstrating their central roles within the module.

Module–trait relationships identified significant correlations between specific modules and traits relevant to pod cracking, including correlations with pectin lyase, cellulose content, hemicellulose content, and soluble pectin content. Further analysis of the Blue, Purple, Magenta, and Red modules provided deeper insights. Gene ontology (GO) analysis highlighted various biological processes such as cell adhesion, unidimensional cell growth, plant-type cell wall organization, vacuolar transport, methylation, pectin biosynthetic process, and lipid transport, with expression patterns indicating downregulation and upregulation across samples (Figure 7A and Table S6). Network visualizations for the top 50 genes from each module, identified using CytoHubba, emphasized the roles of key hub genes. In the Blue module, central genes such as *AKT019250*, *AKT020112*, *AKT002642*, *AKT009693*, and *AKT009091* showed extensive connectivity, crucial for the module’s function (Figure 7B). The Purple module featured hub genes like *AKT006856*, *AKT010061*, *AKT014654*, *AKT009091*, and *AKT002108*, essential for maintaining functional integrity (Figure 7C). In the Magenta module, key genes such as *AKT009091*, *AKT011808*, *AKT019250*, *AKT012999*, and *AKT001629* supported essential biological processes (Figure 7D). The Red module highlighted significant genes like *AKT017106*, *AKT020444*, and *AKT005011*, demonstrating their central roles in the module’s functions (Figure 7E). These findings provide a comprehensive understanding of the functional roles and interactions of genes within these modules, offering potential targets for further studies and genetic manipulation to mitigate pod cracking in Akebia fruits.

## 3. Discussion

**Figure 8:**
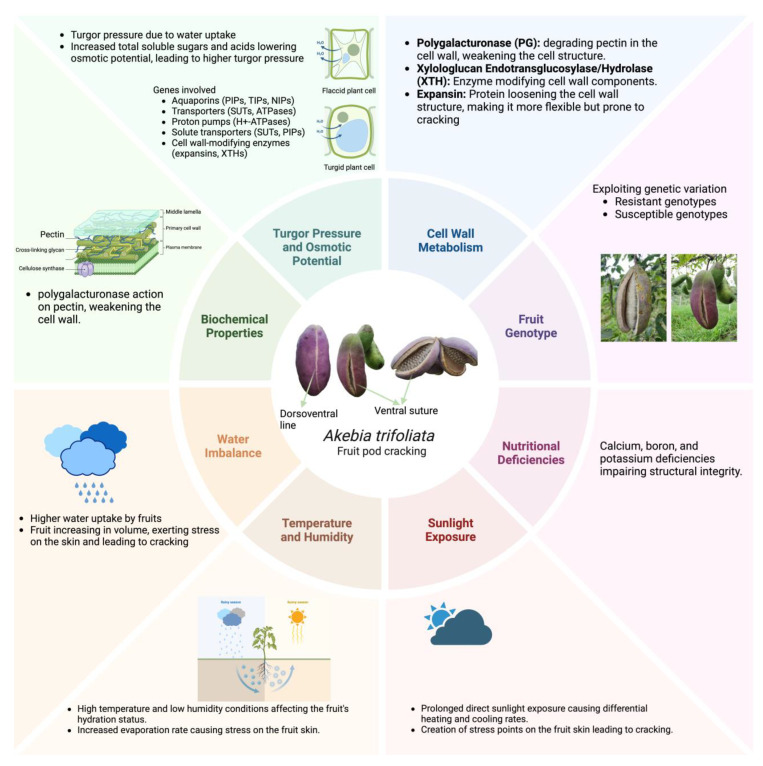
Integrated mechanisms of fruit cracking in *Akebia trifoliata*: the interplay of genetic regulation and environmental factors. This figure illustrates the complex interplay of transcriptional and environmental factors contributing to fruit cracking in *Akebia trifoliata*. The cracking predominantly occurs along the ventral suture, while the dorsoventral line remains intact. Key mechanisms include changes in turgor pressure and osmotic potential, alterations in cell wall metabolism, biochemical properties, water imbalance, temperature and humidity stress, sunlight exposure, nutritional deficiencies, and genetic factors. These elements collectively influence the structural integrity of the fruit, leading to this characteristic pattern of cracking.

Fruit cracking is a widespread and economically significant issue affecting various fruit species [6]. Previous studies have extensively documented the physiological, genetic, and abiotic factors contributing to this phenomenon (Figure 8). For instance, research on sweet cherry and litchi has shown that fruit cracking is closely associated with the weakening of cell wall structures and changes in hormone levels [8,10]. In *A. trifoliata*, cracking predominantly occurs along the ventral suture, while the dorsoventral line remains intact, indicating a specific pattern of structural weakness [11]. This phenomenon is driven by a combination of genetic regulation and environmental stressors [5]. Previous studies primarily focused on the ventral suture [17,18,19]. However, our approach was more comprehensive, sampling both the ventral suture and the dorsoventral line simultaneously to provide a comparative view of changes occurring in fruit structure. This dual sampling provides a broader understanding of fruit cracking mechanisms. Such comprehensive sampling allows for a more detailed exploration of the structural and physiological changes associated with fruit cracking, which is crucial for improving fruit quality and marketability. The research findings emphasize the dynamic changes in cell wall components and enzymatic activities during fruit ripening, highlighting their critical roles in the cracking process.

Our results demonstrate that the ventral suture tissues exhibit significantly elevated enzymatic activities, such as polygalacturonase (PG) and β-galactosidase, compared to the dorsoventral line (DL) tissues. These findings are consistent with previous studies that have shown the involvement of these enzymes in cell wall degradation leading to structural weakening and subsequent fruit cracking [12]. Specifically, the increase in PG and β-galactosidase activities towards the peak ripening period suggests their pivotal roles in facilitating the breakdown of pectin and cellulose, thereby compromising the cell wall integrity in the VS tissues.

The observed differential dynamics in cellulose and hemicellulose contents between VS and DL tissues further corroborate the role of cell wall composition in fruit cracking. Cellulose content peaked early in the VS tissues and declined as ripening progressed, whereas DL tissues maintained relatively stable cellulose levels. This pattern indicates that the mechanical properties of the fruit pericarp, influenced by cellulose accumulation and degradation, are critical determinants of susceptibility to cracking. This aligns with studies on other fruit species where cellulose and pectin dynamics have been implicated in fruit pericarp integrity and cracking resistance [15,16]. A study by Niu et al. [5] on the molecular mechanisms of fruit cracking in *A. trifoliata* provides additional context to our findings. They observed that the cell walls of cracking fruits became thinner and looser, showing substantial breakdown compared to non-cracking fruits. This structural change is consistent with our observation of increased enzymatic activities that degrade cell wall components. Moreover, Niu et al. found that pectate lyases, pectinesterase, and β-galactosidase 2 were significantly upregulated in cracking fruits, indicating their crucial roles in the process [5].

The transcriptomic analysis provided valuable insights into the gene expression profiles associated with fruit cracking. The identification of differentially expressed genes (DEGs) across various developmental stages, particularly in the ventral suture tissues, underscores the significant transcriptional regulation occurring during the ripening process. Notably, genes involved in pectin biosynthesis and catabolism, cell wall organization, and oxidoreductase activities were differentially expressed, indicating their roles in modulating cell wall properties and contributing to cracking. The decrease in the number of differentially expressed genes near the pod cracking stage is likely due to developmental stability and specialization of functions. As pods approach cracking, the developmental processes become more stable, requiring fewer genes to be differentially regulated. Additionally, cells and tissues have specialized, completing most developmental programs, and the regulation of gene expression becomes tightly controlled. This shift towards specific genes involved in cracking, along with the completion of major biosynthetic pathways, results in reduced overall transcriptional activity.

An increase in enzymatic activities such as polygalacturonase (PG) and β-galactosidase near the fruit cracking stage (TP7 and TP8) along with the identification of DEGs (*AKT008327*, *AKT008327*, *AKT015303*, and *AKT022347*) annotated as polygalacturonase suggest their direct involvement in the fruit cracking through cell wall degradation. Previously published reports also suggest an increase in polygalacturonase activity during the fruit ripening and degradation of the cell wall [23,24,25,26,27,28]. Moreover, several expansin genes (*AKT017642*, *AKT017643*, *AKT021517*, *AKT001818*, *AKT017643*, and *AKT017644*) were also identified as DEGs at key stages for fruit cracking. Expansin proteins are crucial for softening and loosening of the cell structure during fruit ripening and development [29,30,31]. For instance, Su et al. demonstrated that expansin and endoglucanase promote fruit softening and cell wall disassembly in tomatoes, and a simultaneous knockdown of both genes increased fruit firmness [29]. Therefore, the above-mentioned genes present significant potential as candidate genes for further functional characterization for pericarp cracking in *A. trifoliata* fruits.

The comprehensive analysis of differentially expressed genes (DEGs) involved in pathways such as pectin biosynthetic and catabolic processes, cell wall organization, oxidoreductase activity, and auxin-activated signaling reveals intricate regulatory mechanisms governing pod cracking in Akebia. The identification of genes like *AKT032945* (pectinesterase) and *AKT045678* (polygalacturonase) underscores the critical role of pectin modification in cell wall loosening and pod dehiscence, consistent with previous studies demonstrating the importance of pectin dynamics in fruit ripening and abscission zones [6,32,33,34]. Furthermore, the differential expression of genes associated with oxidoreductase activity, including multiple peroxidase genes, suggests a potential role of redox homeostasis in regulating cell wall rigidity, which could influence pod cracking susceptibility [5,6,35,36]. The involvement of auxin-related genes, particularly those implicated in the auxin-activated signaling pathway and response to auxin (*AKT022903* and *AKT034567*), highlights the hormone’s significance in coordinating cell growth and separation processes essential for pod opening, aligning with the established roles of auxin in organ abscission [11,37]. For instance, Jiang et al. emphasized the role of auxin-mediated signaling pathway activation during ripening in Akebia fruit [11]. The identified genes, particularly those involved in pectin modification, cell wall organization, and hormonal signaling, such as *AKT032945* (pectinesterase), *AKT014783* (xyloglucan endotransglucosylase), and *AKT012412* (Myb-like DNA-binding domain), represent promising candidates for further functional verification using CRISPR-based gene editing to knock out or induce the overexpression of specific genes and transient expression assays to elucidate their precise roles in the regulatory mechanisms underlying pod cracking.

Weighted gene co-expression network analysis (WGCNA) identified key gene modules correlated with phenotypic traits related to fruit cracking. Modules such as Blue, Purple, Magenta, and Red showed significant associations with traits like pectin lyase activity, cellulose content, and soluble pectin content. The identification of central hub genes within these modules provides potential targets for genetic manipulation aimed at developing crack-resistant *A. trifoliata* varieties.

The findings of this study have several significant implications. First, they enhance our understanding of the molecular basis of fruit cracking in *A. trifoliata*, providing a foundation for future research aimed at improving fruit quality and yield. Second, the identification of key genes and regulatory pathways involved in the cracking process offers potential targets for breeding programs and genetic modifications. By developing crack-resistant varieties, it is possible to reduce post-harvest losses and improve the commercial viability of this underutilized fruit crop.

## 4. Materials and Methods

### 4.1. Plant Materials, Growth Conditions, and Sample Information

*Akebia trifoliata* plants were cultivated under field conditions at the Lushan Botanical Garden, Nanchang, Jiangxi Province, China. The plants were grown under natural sunlight, with a photoperiod of approximately 14 h light and 10 h dark during the growing season. The temperature ranged from 20 °C to 30 °C during the day and from 15 °C to 20 °C at night. Relative humidity was maintained between 60% and 80%. Regular irrigation and fertilization practices were followed to ensure optimal growth conditions. Plants were spaced 1.5 m apart within rows, with rows spaced 2 m apart.

Fruit samples were collected at nine different developmental stages during the ripening phase from July to September. The sampling dates were July 2, August 3, September 1, September 20, September 25, September 30, October 5, October 10, and October 25 (Table 1). Fruits were sampled from the ventral suture (VS) and dorsoventral line (DL) regions, labeled as F and B tissues, respectively. Each developmental stage had three biological replicates, with samples immediately frozen in liquid nitrogen and stored at −80 °C for further analysis.

### 4.2. Physiological Trait Analysis

To investigate the physiological changes associated with fruit cracking, various enzymatic activities and cell wall constituents were measured in triplicate.

Polygalacturonase (PG): Activity was measured using a colorimetric assay based on the release of reducing sugars from polygalacturonic acid. The reaction mixture was incubated at 37 °C for 1 h, and the reducing sugars were quantified using the DNS method.

β-Galactosidase (β-GAL): Activity was determined using a p-nitrophenyl-β-D-galactopyranoside (PNPG) substrate, with the release of p-nitrophenol measured spectrophotometrically at 405 nm after incubation at 37 °C for 30 min.

Pectin Lyase (PL): Activity was assayed by measuring the increase in absorbance at 235 nm due to the formation of unsaturated products from pectin. The reaction was carried out at 30 °C for 30 min.

Cellulase (Cx): Activity was assessed using carboxymethyl cellulose as a substrate. The release of reducing sugars was measured using the DNS method after incubating the reaction mixture at 50 °C for 1 h.

Cell Wall Constituents:

Cellulose Content (CC): This was determined using the Updegraff method, where cellulose is hydrolyzed, and the resulting glucose is quantified. Samples were treated with acetic/nitric reagent, and the cellulose was hydrolyzed with sulfuric acid.

Hemicellulose Content (HC): This was measured after sequential extraction of cell wall material with 1 M potassium hydroxide, followed by quantification of pentose and hexose sugars using the orcinol method.

Total Pectin Content (TPC): This was extracted using acidified water, followed by quantification using the carbazole–sulfuric acid method. The reaction was incubated at 80 °C for 20 min before measuring absorbance at 525 nm.

Soluble Pectin Content (SPC): This was measured similarly to TPC, but using a water-soluble extraction method at room temperature.

### 4.3. RNA Extraction and Transcriptome Sequencing

Total RNA was extracted from F and B tissues using the TRIzol reagent (Invitrogen Waltham, Massachusetts, U.S) following the manufacturer’s instructions. RNA quality and concentration were assessed using a NanoDrop spectrophotometer and agarose gel electrophoresis. RNA integrity was evaluated using an Agilent Bioanalyzer, and only samples with an RNA Integrity Number (RIN) above 7.0 were used. High-quality RNA samples were used for cDNA library construction and sequencing on an Illumina HiSeq 2500 platform. Clean reads were obtained by removing low-quality reads and adapters using FastQC and Trimmomatic version 0.40.

### 4.4. Differential Gene Expression Analysis

Transcriptome data were analyzed to identify differentially expressed genes (DEGs) between the ventral suture and dorsoventral line at various developmental stages. DESeq2 version 3.20 was used to determine DEGs with a false discovery rate (FDR) < 0.05. Gene ontology (GO) enrichment analysis was performed to identify biological processes significantly enriched among DEGs using the GOseq R package version 3.20.

### 4.5. Weighted Gene Co-Expression Network Analysis (WGCNA)

Weighted gene co-expression network analysis (WGCNA) was conducted to identify modules of highly correlated genes. An appropriate soft threshold power was selected to achieve a scale-free topology. Module–trait relationships were analyzed to identify significant correlations between module eigengenes and phenotypic traits. Key hub genes within significant modules were identified using the CytoHubba plugin in Cytoscape version 3.10.3.

### 4.6. Statistical Analysis

All physiological and enzymatic data were analyzed using a two-way ANOVA to assess the effects of tissue type and developmental stage. Tukey’s HSD post hoc test was performed following an ANOVA to identify specific differences between groups. Principal component analysis (PCA) and correlation matrices were constructed to visualize the relationships among the traits. Physiological traits were standardized (mean-centered and scaled to unit variance) before performing PCA to ensure comparability across different scales. Statistical analyses were performed using R software version 4.4, with significance set at *p* < 0.05.

## 5. Conclusions

In conclusion, this study highlights the critical roles of cell wall components, enzymatic activities, and gene expression in the cracking process of *A. trifoliata* fruits. The integration of physiological, biochemical, and transcriptomic analyses provides a comprehensive understanding of the factors contributing to fruit cracking. Future research should focus on validating the identified candidate genes and exploring their functional roles in fruit cracking, ultimately leading to the development of improved cultivation and breeding strategies for *A. trifoliata*.

## Figures and Tables

**Table 1:** Sample information.

Sampling Date	Sample ID	Developmental Stages of Ventral Suture	Developmental Stages of Dorsoventral Line	Status
20 July 2023	TP1	FA	BA	uncracked
30 August 2023	TP2	FB	BB	uncracked
10 September 2023	TP3	FC	BC	uncracked
20 September 2023	TP4	FD	BD	uncracked
25 September 2023	TP5	FE	BE	uncracked
30 September 2023	TP6	FF	BF	uncracked
5 October 2023	TP7	FG	BG	uncracked
10 October 2023	TP8	FH	BH	started to crack
15 October 2023	TP9	FJ	BJ	cracked

F and B indicate the ventral suture and dorsoventral line, respectively, while A–H and J indicates time-dependent developmental stages of fruit ripening in *Akebia trifoliata.*

## Data Availability

Data will be made available upon request.

## Supplementary Materials

The following supporting information can be downloaded at https://www.mdpi.com/article/10.3390/ijms252212388/s1.
